# Practical steps to improving the management of type 1 diabetes: recommendations from the Global Partnership for Effective Diabetes Management

**DOI:** 10.1111/j.1742-1241.2009.02296.x

**Published:** 2010-02

**Authors:** P Aschner, E Horton, L A Leiter, N Munro, J S Skyler

**Affiliations:** 1 Javeriana University School of MedicineBogota, Colombia; 2 Joslin Diabetes CenterBoston, MA, USA; 3 St Michael’s Hospital and University of TorontoToronto, ON, Canada; 4 Beta Cell Diabetes Centre, Chelsea and Westminster HospitalLondon, UK; 5 University of Miami and Diabetes Research InstituteMiami, FL, USA

## Abstract

The Diabetes Control and Complications Trial (DCCT) led to considerable improvements in the management of type 1 diabetes, with the wider adoption of intensive insulin therapy to reduce the risk of complications. However, a large gap between evidence and practice remains, as recently shown by the Pittsburgh Epidemiology of Diabetes Complications (EDC) study, in which 30-year rates of microvascular complications in the ‘real world’ EDC patients were twice that of DCCT patients who received intensive insulin therapy. This gap may be attributed to the many challenges that patients and practitioners face in the day-to-day management of the disease. These barriers include reaching glycaemic goals, overcoming the reality and fear of hypoglycaemia, and appropriate insulin therapy and dose adjustment. As practitioners, the question remains: how do we help patients with type 1 diabetes manage glycaemia while overcoming barriers? In this article, the *Global Partnership for Effective Diabetes Management* provides practical recommendations to help improve the care of patients with type 1 diabetes.

**What's known**

- Considerable gaps in the care of adult patients with type 1 diabetes remains, with a high proportion of patients developing diabetes-related complications.
- The majority of patients do not achieve glycaemic goals because of barriers related to insulin dose adjustment, self-monitoring of blood glucose and fear of hypoglycaemia.
- Living with diabetes is not easy, and achievement of glycaemic goals requires the patient to undertake self-care behaviours with relentless vigilance.
- Structured education supported by a multidisciplinary team approach can play an integral role in helping overcome these barriers.

**What's new**

- This article provides guidance on where gaps in care remain and how to address them based on recent evidence.

## Introduction: current challenges in type 1 diabetes

Diabetes affects 246 million people worldwide and, of these, approximately 22 million adults and 0.4 million children have type 1 diabetes (1). The impact of diabetes-related complications on patients and healthcare systems is significant, with reported cumulative incidences of proliferative retinopathy, nephropathy and cardiovascular disease (CVD) of 47%, 17% and 14%, respectively, after 30 years of diabetes (2).

Type 1 diabetes is an autoimmune disease, in which environmental factors are thought to trigger the autoimmune destruction of pancreatic β-cells in genetically susceptible individuals. Although great progress has been made to date in identifying genetic markers (3,4), widespread genetic screening for susceptibility to the disease is not yet possible. In young adults, there is evidence that the onset of type 1 diabetes may be progressive and characterised by a slower decline in β-cell function compared with children and adolescents (5). Importantly, data from the Diabetes Control and Complications Trial (DCCT) suggest that residual β-cell function is associated with improved outcomes, such as better glycaemic control and lower risk for hypoglycaemia and chronic complications (6).

Evidence also shows that optimisation of glycaemic control at an early stage significantly reduces the risk of microvascular and macrovascular complications, as clearly demonstrated in the DCCT and its long-term follow-up study, the Epidemiology of Diabetes Interventions and Complications (EDIC) trial (7–9). Despite the clear benefits of intensive glycaemic control, there is still a large gap between evidence and practise, with the majority of patients not reaching targets. In the recent DCCT-EDIC/EDC analysis, 81–87% of patients had an HbA_1c_ > 7.0% (2), which is consistent with the UK findings of up to 74% of patients with HbA_1c_ > 7.5% (10). There are a number of barriers to glycaemic control in type 1 diabetes, including the occurrence and fear of hypoglycaemia and the complexity and demands of day-to-day management, in particular the need for frequent self-monitoring of blood glucose (SMBG) and regular adjustments in insulin dosing. These challenges have an enormous impact on patient quality of life and healthcare costs are also considerable (11). In the future, we hope to be able to prevent this condition with advances in transplantation techniques or new agents. However, for practitioners involved in diabetes care at this time, the question remains: how do we help patients with type 1 diabetes to better manage glycaemia to reduce complications and improve quality of life? To facilitate this, we must be able to translate what we have learned in the clinical trial setting to the clinic and it is this approach that underlies the recommendations in this article.

The *Global Partnership for Effective Diabetes Management* is a multidisciplinary group of health practitioners from leading health institutions and research organisations around the world. Since 2004, the main remit of our group has been to facilitate improvements in diabetes care through educational initiatives. While our previous publications have focused on type 2 diabetes (12,13), we recognise the considerable overlap, as well as important differences, between optimal patient management practices in type 1 and type 2 diabetes. We have therefore broadened our scope to provide practical guidance on the day-to-day management of patients with type 1 diabetes, with key recommendations summarised in Table 1. Because of the wide range of issues faced by patients with type 1 diabetes, this article will focus specifically on adult care. While the implementation of some recommendations may not be possible in all regions, we hope this article serves as a benchmark for the management of all patients with type 1 diabetes.

**Table 1:** Practical recommendations for the management of adults with type 1 diabetes

Aim for as good glycaemic control as possible while minimising the risk of hypoglycaemia.
Ensure regular and appropriate monitoring for complications.
Initiate an intensive basal-bolus insulin regimen as early as possible.
Provide all patients with a structured educational programme at initiation of insulin and thereafter.
Ensure that self-monitoring is universally adopted as an integral part of insulin therapy.
Provide education about prevention, recognition and treatment of hypoglycaemia at initiation of insulin therapy and thereafter.
Manage all cardiovascular risk factors.
Explore psychological issues associated with type 1 diabetes and treat/refer as appropriate.
Adopt a multidisciplinary team approach with shared goals and recommendations.

## Managing hyperglycaemia in type 1 diabetes

### Early optimisation of glycaemic control

**Figure 1:**
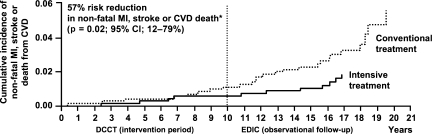
DCCT/EDIC: Cumulative incidence of any CVD event with intensive vs. conventional insulin treatment in patients with type 1 diabetes (*n*= 1397) (8). MI, myocardial infarction. *Intensive vs. conventional treatment. Copyright © 2005 Massachusetts Medical Society. All rights reserved.

Optimisation of glycaemic control at an early stage of the disease is the most fundamental aspect of care in type 1 diabetes for preventing microvascular and macrovascular complications, as shown in the pivotal DCCT/EDIC study. In the DCCT, patients with type 1 diabetes randomised to intensive therapy (≥ 3 insulin injections per day or pump therapy) had tighter glycaemic control than those who received conventional treatment (1–2 insulin injections per day) (mean HbA_1c_: 7.1% vs. 9.1%, respectively) (7). Intensive treatment significantly reduced the incidence of retinopathy by 76%, the progression of retinopathy by 54%, the development of proliferative or severe non-proliferative retinopathy by 47%, the occurrence of microalbuminuria by 39%, of nephropathy by 54% and of clinical neuropathy by 60% (7). The difference in HbA_1c_ between the groups accounts for > 90% of the benefit associated with intensive therapy (14). Later, with extended follow up, DCCT/EDIC showed that non-fatal myocardial infarction, stroke or death from CVD was reduced by 57% and occurrence of any CVD event was reduced by 42% (Figure 1) (8).

**Figure 2:**
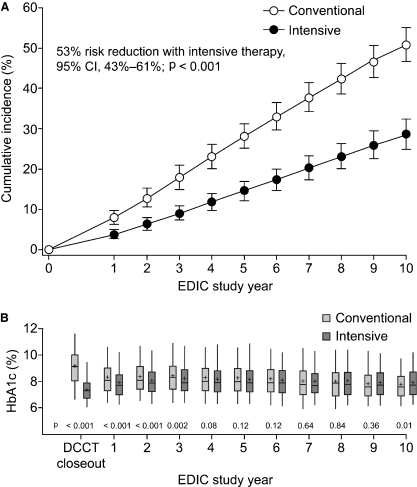
EDIC study: (A) Cumulative incidence of retinopathy (*n*= 1349) and (B) HbA_1c_ values (*n*= 1211) over 10 years after the DCCT trial in which patients with type 1 diabetes were treated with intensive vs. conventional insulin therapy (9). (A) Error bars are 95% confidence intervals. (B) Box presents quartiles of distribution; vertical lines show the 95th and 5th percentiles; horizontal line is median; + indicates mean. Copyright © 2008 American Medical Association. All rights reserved.

The need to optimise glycaemic control as early as possible is also supported by the ‘metabolic memory’ or ‘legacy effect’ observed in DCCT/EDIC, where the benefit of intensive glycaemic control on the risk of complications was found to endure even after HbA_1c_ levels subsequently increased. For example, the reduced risk of retinopathy associated with intensive insulin therapy persisted for up to 10 years in the observational follow-up EDIC study, despite the convergence of HbA_1c_ levels in the intensive and conventional groups (HbA_1c_∼8.0%; Figure 2) (9).

Despite the known benefits of glycaemic control, many patients do not reach glycaemic targets (Table 2), with hypoglycaemia or even fear of hypoglycaemia being one of the major barriers. Other barriers include the complexity of some regimens, causing some patients to regularly omit insulin. Additional obstacles include poor adherence to treatment, lack of social support and lack of access to a specialist care centre, as well as psychological barriers (15), which are discussed later in this article. Individuals with poor glycaemic control are at an increased risk of complications and should therefore be aiming for as good glycaemic control as possible.

**Table 2:** Glycaemic targets for individuals with type 1 diabetes

	ADA (16)	CDA (17)	IDF (51)	NICE (UK) (18)
HbA_1c_	< 7.0%	≤ 7.0%	6.2–7.5%	≤ 6.5–7.5%
Fasting preprandial glucose, mg/dl (mmol/l)	70–130 (3.9–7.2)	72–126 (4.0–7.0)	91–120 (5.1–6.5)	72–144 (4.0–8.0)
Postprandial glucose, mg/dl (mmol/l)	< 180* (< 10.0)	90–180† (5.0–10.0)	136–160‡ (7.6–9.0)	< 180‡ (< 10.0)

ADA, American Diabetes Association; CDA, Canadian Diabetes Association; IDF, International Diabetes Federation; NICE, National Institute for Health and Clinical Excellence.

The CDA guidelines note that HbA_1c_ goals and strategies must be tailored to the individual with diabetes, with consideration given to individual risk factors.

ADA and CDA glycaemic targets are for type 1 and type 2 diabetes.

* Peak postprandial capillary plasma glucose.

† 90–144 mg/dl (5.0–8.0 mmol/l) if HbA_1c_ target not being met.

‡ Capillary postprandial glucose 1–2 h after meal.

***Recommendation: Aim for as good glycaemic control as possible while minimising the risk of hypoglycaemia*.**

**Figure 3:**
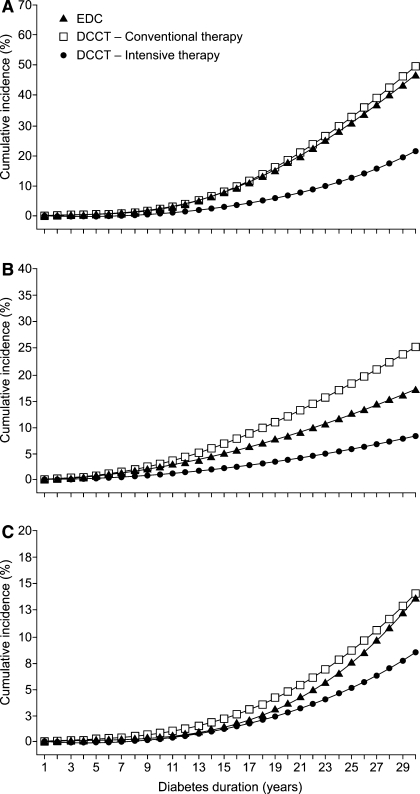
Cumulative incidences of (A) proliferative retinopathy or worse, (B) nephropathy and (C) CVD over time in the DCCT intensive therapy group, DCCT conventional therapy group and EDC cohort (2). Nephropathy was defined as albumin excretion rate ≥ 300 mg/24 h, serum creatinine ≥ 2 mg/dl, or dialysis or renal transplant. CVD was defined as: non-fatal myocardial infarction or stroke, CVD death, subclinical myocardial infarction, angina, angioplasty or coronary artery bypass. Copyright © 2009 American Medical Association. All rights reserved.

DCCT/EDIC showed not only the importance of early glycaemic control for the prevention of complications, but that it can slow the progression of complications. For example, in patients with retinopathy at baseline in the DCCT, intensive insulin therapy slowed progression by 54% (7). The benefits of early optimisation of glycaemic control were most recently demonstrated in the combined analysis of data from DCCT/EDIC and the ‘real world’ observational EDC study (2). After 30 years of diabetes, the cumulative incidences of proliferative retinopathy, nephropathy and CVD were substantially lower in the DCCT intensive therapy group (21%, 9% and 9% respectively) compared with the DCCT conventional group (50%, 25% and 14%) or the EDC cohort (47%, 17% and 14%) (Figure 3) (2). (HbA_1c_ values in the EDC cohort were 9.0–9.3% until year 8 and fell by ∼0.5% thereafter.) In light of these data, it is clearly important to identify complications and associated risk factors as early as possible so that they may be managed appropriately and effectively. It may be necessary to reorganise clinical systems to ensure that regular surveillance for complications becomes a routine part of care. Many guidelines recommend annual screening for microvascular and macrovascular complications in adults with type 1 diabetes (16–18). Recent advice published by the American Diabetes Association is shown in Table 3 (16). If complications are present, interventions to reduce the risk of progression should be implemented as soon as possible and patients referred to specialist care as appropriate.

**Table 3:** Screening for complications in adults with type 1 diabetes; recommendations from the American Diabetes Association (16)

Care	Screening
Retinopathy	Refer for an initial dilated and comprehensive eye examination within 5 years after diabetes onset and annually thereafter. Consider less frequent examination (every 2–3 years) following one or more normal eye examinations. More frequent examinations required if retinopathy is progressive.
Chronic kidney disease	Perform an annual urine albumin excretion test in patients with type 1 diabetes of ≥ 5 years’ duration. Measure serum creatinine at least annually, regardless of degree of urine albumin excretion.
Neuropathy	Screen all patients for distal symmetrical polyneuropathy at diagnosis and at least annually thereafter using simple clinical tests such as pinprick sensation, vibration perception (using a 128 Hz tuning fork), 10 g monofilament pressure sensation at the distal plantar aspect of both great toes and metatarsal joints, and assessment of ankle reflexes. Institute screening for signs and symptoms of cardiovascular autonomic neuropathy 5 years after diagnosis of type 1 diabetes.
Dyslipidaemia	Measure fasting lipid profile at least annually in most adult patients. Aggressively treat lipid and blood pressure abnormalities.

***Recommendation: Ensure regular and appropriate monitoring for complications*.**

## Insulin therapy

### Initiation of insulin

Intensive insulin therapy using a basal-bolus approach, whether as multiple daily injections or pump therapy, is considered the best treatment for individuals with type 1 diabetes regardless of age (16,17,19). This is because it provides greater glycaemic control and has been shown to reduce the risk of complications compared with conventional fixed-dose regimens (7,8), most recently shown in the DCCT/EDIC-EDC analysis (2). While achieving good glycaemic control is important in all age groups, it is of particular importance in children with type 1 diabetes, as they face the longest duration of the disease. Generally available insulin preparations are summarised in Table 4, along with their pharmacokinetic profiles. The choice of insulin and mode of delivery should be guided by factors such as age, lifestyle, general health, motivation, ability for self-management and diet, as well as availability/accessibility (17). Consideration should be given to risk of hypoglycaemia with, for example, longer-acting insulin analogues (glargine and detemir) being preferable to intermediate-acting human insulin [neutral protamine Hagedorn (NPH)], as they are associated with reduced risk of hypoglycaemia (20–25), including nocturnal hypoglycaemia (24). Rapid-acting insulin analogues (lispro, aspart and glulisine) may be preferred over regular human insulin, as they have been associated with greater improvements in HbA_1c_ with reduced risk of hypoglycaemia (26,27).

**Table 4:** Pharmacokinetics of human insulin and analogues (87) (may depend on local availability)

Class of insulin	Formulation	Onset (min)	Peak (h)	Duration (h)
Basal insulins				
Long-acting analogues	Insulin glargine	66	–	Up to 24
	Insulin detemir	48–120	–	Up to 24
Intermediate-acting human	NPH, human	60–120	6–14	16–24
Prandial insulins				
Rapid-acting analogues	Insulin lispro	15–30	0.5–2.5	3–6.5
	Insulin aspart	10–20	1–3	3–5
	Insulin glulisine	10–15	1–1.5	3–5
Short-acting human	Regular, human	30–60	1–5	6–10
Premixed insulins				
Premixed analogues	BiAsp 70/30	10–20	1–4	Up to 24
	Insulin lispro 75/25	15–30	1–6.5	Up to 24
	Insulin lispro 50/50	15–30	0.75–13.5	Up to 24
Premixed human	70% NPH/30% regular	30–60	2–16	Up to 18–24

BiAsp, biphasic insulin aspart; NPH, neutral protamine Hagedorn.

A recent meta-analysis found that glycaemic control was significantly better with continuous subcutaneous insulin infusion (CSII) compared with multiple daily injections in adults with type 1 diabetes who had severe hypoglycaemia, with a difference in HbA_1c_ of 0.62% (28). The study also reported a threefold reduction in severe hypoglycaemia with CSII compared with multiple daily injections. Patients with the highest rates of initial severe hypoglycaemia had the greatest reduction in events. Thus, CSII using a pump device may be preferable over multiple injections in some patient groups, such as those with poor glycaemic control and individuals experiencing frequent severe hypoglycaemia or requiring greater lifestyle flexibility (28–30). Insulin pumps also have a number of practical advantages which can mean greater lifestyle flexibility for the patient in terms of dietary freedom, activity and a general improvement in quality of life. However, one of the main drawbacks of pump therapy is cost, which is a barrier for many patients worldwide.

There may be a misconception that intensive insulin therapy is not necessary at diagnosis and particularly in the ‘honeymoon period’ because of the residual β-cell function/insulin secretion that persists months after diagnosis in some individuals with type 1 diabetes. However, studies such as the DCCT have clearly established that intensive therapy should be initiated as soon as possible to prevent diabetes-related complications (8,9) and preserve β-cell function (6). This approach should be the case in the great majority of individuals, regardless of the mode of insulin delivery used.

***Recommendation: Initiate an intensive basal-bolus insulin regimen as early as possible*.**

### Insulin dose adjustment

It is important that patients adjust their insulin doses appropriately in response to factors such as carbohydrate intake, lifestyle, exercise and intercurrent illness to minimise the risk of hypo- or hyperglycaemia. As described above, insulin pumps may allow greater flexibility of dosing, but as all patients will not have access to pump therapy, alternative strategies are needed and education is required for all patients.

Modification of insulin dosages based on diet and exercise can be challenging for patients and should be considered an essential part of patient education. Structured education programmes have been demonstrated to have substantial benefits in terms of outcomes (31–35). For example, the UK-based Dose Adjustment For Normal Eating (DAFNE) programme has been shown to improve glycaemic control and quality of life while saving costs, without increasing the risk of severe hypoglycaemia (34,35). Similarly, in the Dusseldorf Diabetes Treatment and Teaching Programme (DTTP), which involves a 5-day inpatient course for individuals with type 1 diabetes, HbA_1c_ fell significantly from 8.1% to 7.3% over the subsequent year, as did the number of severe hypoglycaemic episodes (0.37 vs. 0.14 events per patient-year) (31). These types of programmes demonstrate that appropriate education can improve glycaemic control while giving the individual more flexibility in terms of diet rather than having to adhere to rigid calorie control and fixed insulin doses. Other important considerations include the use of carbohydrate counting, a common meal-planning method used by patients, which must of course be adapted to local diet and lifestyle. Appropriate adjustment of insulin doses surrounding exercise is also important. Despite the well-known health benefits of exercise, 64% of patients with type 1 diabetes do not achieve recommended physical activity levels because of barriers such as fear of hypoglycaemia (36,37). In addition, many patients may not know the effect of factors such as exercise or alcohol on glucose levels and the need for appropriate adjustment of insulin therapy, highlighting the importance of education on this subject.

Another consideration is how to adjust insulin dosages during intercurrent illness. In some cases, patients may cease taking insulin altogether, particularly if they are unable to ingest food. This can lead to serious metabolic derangements including diabetic ketoacidosis (DKA). In patients hospitalised for DKA, inadequate insulin dosing was found to be the identifiable cause of DKA in up to 45% of cases (38). It is also important that patients are aware that infection generally exacerbates hyperglycaemia and, thus, they should monitor their glucose levels and continue to take insulin as appropriate, even if their caloric consumption is reduced. Ideally, these types of issues should be addressed as part of appropriate patient self-management education and reiterated during regular reviews.

***Recommendation: Provide all patients with a structured educational programme at initiation of insulin and thereafter.***

### Self-monitoring of blood glucose

Self-monitoring of blood glucose is so fundamental that insulin therapy should always comprise insulin therapy plus SMBG. Patients should receive appropriate training in SMBG when insulin therapy is initiated and periodically thereafter. Self-monitoring provides patients with immediate feedback of the effects of insulin dosage and timing, diet, exercise and stress on glucose levels, providing valuable information on pre- and postprandial and nocturnal glucose levels (39). In addition, self-monitoring should be supported by the diabetes team through discussion of results with patients during each clinic visit to help improve the efficacy and safety of insulin therapy. It should also be emphasised to patients that self-monitoring is not an end in itself, but that the results should be acted on. Clear guidance should be given to patients as to how to adjust insulin dose in response to their results. This should include advice on how to avoid overcorrection when, for example, patients administer an inappropriately large dose of rapid-acting insulin in response to high blood glucose levels.

Patients should monitor glucose levels at least three times per day or more (16–18) and testing should include pre- and postprandial measurements (40). Self-monitoring allows patients to adjust insulin doses based on day-to-day requirements, depending on factors such as activities and meals. Karter et al. found adults with type 1 diabetes who self-monitored three or more times per day had an HbA_1c_ 1% lower than patients who monitored less frequently or not at all (41). More frequent monitoring should be considered in certain circumstances, such as hypo- or hyperglycaemic symptoms, hypoglycaemia unawareness, intercurrent illness, gastroparesis, pregnancy, brittle diabetes or rigorous physical activity (39).

Despite the clear benefits of regular monitoring, SMBG places complex behavioural demands on patients, and up to 64% of individuals do not regularly self-monitor (2). There are a range of barriers to self-monitoring, including patient motivation, psychological barriers, cost, socioeconomic status and education level (39,42). It is important that patients learn to overcome these barriers and are provided with appropriate support to do so. For example, cost is a difficult barrier to overcome, but there is evidence that providing patients with free testing strips improves glycaemic control and compliance with self-monitoring (43).

***Recommendation: Ensure that self-monitoring is universally adopted as an integral part of insulin therapy*.**

Recent progress has led to the development of continuous glucose monitoring (CGM) (44–48), which appears to have certain benefits in regions where it is available. Studies in adults with poorly controlled type 1 diabetes (HbA_1c_≥ 7.0%) have shown a significant reduction in HbA_1c_ (−0.5%) with CGM compared with SMBG over 26 weeks, without an increase in hypoglycaemia (46,48). These effects were sustained for up to 1 year (48). Furthermore, in adults with well-controlled type 1 diabetes using CGM, HbA_1c_ levels were maintained at baseline values (6.4%), with less hypoglycaemia, whereas HbA_1c_ rose (from 6.5% to 6.8%) in patients who used SMBG over 26 weeks (47). CGM also limits glycaemic excursions (45). However, CGM is not currently appropriate for all patients, although this may change in the future. Moreover, a closed loop or partially closed loop system including CGM in tandem with an insulin pump may become available (49).

### Hypoglycaemia

Hypoglycaemia is a common problem in type 1 diabetes that can affect all aspects of life including personal relationships, employment, driving, physical activity and travel (50). The degree of hypoglycaemia can vary hugely, from no symptoms to a serious life-threatening condition. Fear of hypoglycaemia in both patient and physician can prevent individuals from achieving optimal glycaemic control and can have a major impact on quality of life. Guidelines generally define hypoglycaemia as plasma glucose < 4.0 mmol/l (< 72 mg/dl) (16,17,51). However, patients may not consider low blood glucose levels as a sign of hypoglycaemia if they are asymptomatic; thus, the importance of monitoring and keeping blood glucose levels above this threshold, regardless of presence or absence of symptoms, should be emphasised.

**Figure 4:**
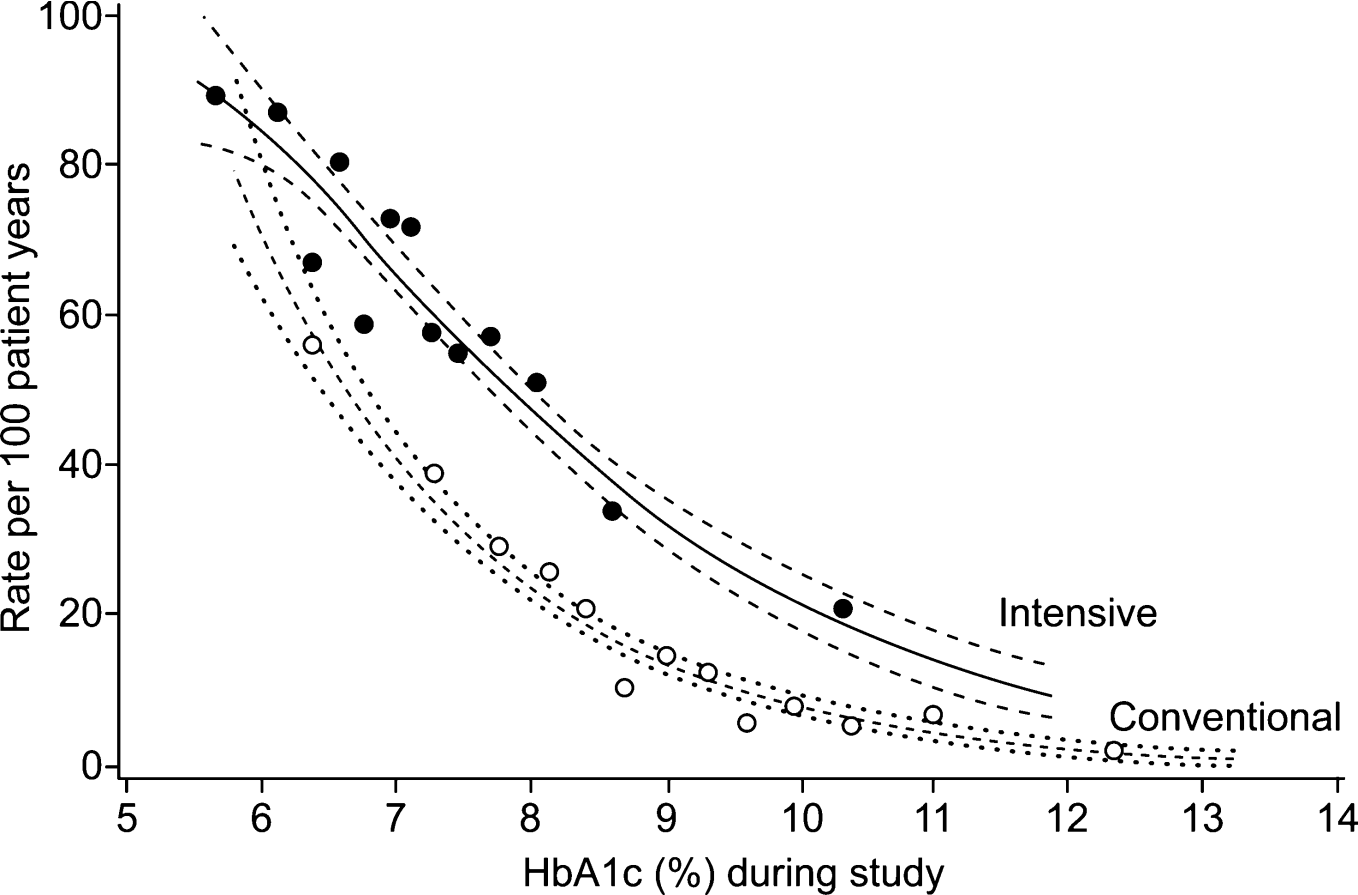
Risk of severe hypoglycaemia vs. HbA_1c_ in the intensive (•) and conventional (○) groups during the DCCT (*n*= 1441) (53). Copyright © 1997 American Diabetes Association. Reprinted with permission from The American Diabetes Association.

In the DCCT, severe hypoglycaemia was three times higher with intensive therapy compared with conventional therapy (Figure 4) (52,53), although the actual frequency may be even higher outside the clinical trial setting (54). Yet, it should be appreciated that the absolute frequency of severe hypoglycaemia may be lower with the use of analogue insulin therapy. Patients who experience severe hypoglycaemia are at increased risk of subsequent episodes, with almost one-third experiencing a second episode within 4 months (53). Nocturnal hypoglycaemia is also a significant health burden, with almost half of severe hypoglycaemic episodes occurring at night (52). Risk factors associated with hypoglycaemia are numerous but include strict glycaemic control (HbA_1c_ < 6.0%), prior episode of severe hypoglycaemia, longer duration of diabetes, autonomic neuropathy and hypoglycaemia unawareness (17,50,53). Of note, in the DCCT, intensively treated patients with greater residual β-cell function (C-peptide 0.21–0.5 nmol/l) had a significantly lower rate of hypoglycaemia compared with those with less or no residual β-cell function (0.07 vs. 0.16–0.21 events per patient-year) (55).

Overall, it is imperative that patients receive appropriate information about hypoglycaemia, which should take place upon diagnosis of diabetes and regularly thereafter at follow-up consultations. As mentioned above, SMBG can provide valuable information, and health professionals and patients should be as vigilant of low glucose as high glucose levels.

Patients should be reminded to avoid behaviours that can contribute to hypoglycaemia, such as taking excess insulin, delaying or missing meals, etc. In addition, not only is mis-timing insulin/food intake around exercise a risk for hypoglycaemia (52) but patients are often unaware that hypoglycaemia can occur for up to 12 h after exercise; they should therefore be advised on appropriate action including additional monitoring before bed and appropriate increased food intake. Alcohol consumption can also lead to hypoglycaemia and impair recovery from a hypoglycaemic episode; the importance of not omitting food when drinking should be emphasised to patients. Additionally, patients should be advised to have a glucagon emergency kit on hand for severe hypoglycaemic episodes.

***Recommendation: Provide education about prevention, recognition and treatment of hypoglycaemia at initiation of insulin therapy and thereafter*.**

Hypoglycaemia unawareness is an important consideration, as it can increase the risk of severe hypoglycaemia sixfold (50). A retrospective survey of individuals with type 1 diabetes suggests that as many as 20% of patients may be affected (56). In these individuals, changes in the symptom profile may hamper the recognition of impending hypoglycaemia; for example, neuroglycopenic symptoms (poor concentration, drowsiness and difficulties in speech and physical coordination) become more prominent, whereas autonomic symptoms (anxiety, palpitations, sweating and hunger) are blunted or even absent (50). In terms of education, those with hypoglycaemia unawareness will require additional help in recognising its onset, as signs and symptoms are altered. Enabling patients to avoid mild hypoglycaemia can subsequently improve awareness (57,58).

### Managing cardiovascular risk factors

Although the link between type 2 diabetes and CVD is well-known, the increased risk of CVD in type 1 diabetes may be overlooked. However, men with type 1 diabetes have a 3.6-fold higher risk of CVD and women a 7.7-fold higher risk than those without diabetes (59). Furthermore, men with type 1 diabetes aged 45–55 years have the same absolute risk of CVD as men ∼10–15 years older without diabetes, with an even greater difference in women (59).

There is debate concerning the question of whether type 1 diabetes is itself a risk factor for CVD. Evidence suggests that the increase in CVD risk in type 1 diabetes is largely associated with nephropathy (60–64). Indeed, it has been proposed that microalbuminuria and CVD may share common pathophysiological processes, such as endothelial dysfunction and chronic low-grade inflammation (65). Furthermore, data from a large type 1 diabetes cohort have shown that relative mortality from CVD was 37 times greater in those with proteinuria compared with the general population, whereas CVD mortality was only 4.2 times greater in those without proteinuria compared with the general population (66).

However, other risk factors are also present in type 1 diabetes, including hyperglycaemia itself, as shown in the DCCT/EDIC (8,67). It is notable that the metabolic syndrome is becoming more prevalent in the type 1 diabetes population (68) and is associated with an additional 2.5-fold increased risk of cardiovascular and diabetes-related mortality (adjusted for traditional risk factors and diabetic nephropathy) (69). Lifestyle factors may also play a role: adults with long-standing type 1 diabetes have been found to consume a high-fat atherogenic diet compared with those without diabetes (70).

Given the increased risk of CVD, it is apparent that more could be done to address cardiovascular risk factors in type 1 diabetes. In the Pittsburgh Epidemiology of Childhood-Onset Diabetes Complications Study, the event rate of coronary artery disease did not decline over the 30-year follow-up period (1950–1980), despite significant reductions over time in other complications such as renal failure and neuropathy (71). Additional analyses indicated inadequate management of cardiovascular risk factors in this population, with sub-optimal control of hypertension in 72% and of hypercholesterolaemia in 94% of patients (72). Although there is generally a lack of data from large prospective studies of cardiovascular medications in type 1 diabetes, statin therapy has been shown to be as effective in type 1 diabetes as in type 2 diabetes and should be considered in patients with diabetes at sufficiently high risk of vascular events (73). Overall, it is important to monitor and manage cardiovascular risk factors in patients with type 1 diabetes as appropriate. While data are limited, patients with type 1 diabetes of a duration of at least 15 years and over 30 years of age should be considered at high risk of CVD (17).

***Recommendation: Manage all cardiovascular risk factors*.**

### Psychological aspects of the disease

It is important for practitioners to be aware not only of the heavy burden that patients with type 1 diabetes face in terms of practical day-to-day management but also of the significant psychological impact of the disease. Recent evidence suggests that the prevalence of depression and anxiety symptoms is considerably higher in patients with type 1 diabetes compared with the general population (74,75). The consequences of psychiatric disorders in type 1 diabetes are far-reaching and are associated with hyperglycaemia and treatment non-adherence as well as with the long-term complications of the disease (76–83). Some groups may be more vulnerable to psychiatric problems, such as teenage girls and women who are particularly prone to eating disorders and may omit insulin doses as a means of weight control (84,85), which can adversely affect optimal management and outcomes (86) in type 1 diabetes.

Guidelines recommend that psychological screening should generally be a routine part of diabetes management (16–18). The overall challenge for all members of the multidisciplinary team is to be aware of and able to recognise the psychological impact of the disease and to refer patients to specialist care when appropriate. As mentioned above, a psychologist or psychiatrist should be considered part of the multidisciplinary team wherever possible.

***Recommendation: Explore psychological issues associated with type 1 diabetes and treat/refer, as appropriate*.**

## A team approach to diabetes care

As described above, there are many complexities involved in treating patients with type 1 diabetes and helping them to achieve and maintain their glycaemic targets. Therefore, adopting a team approach that involves a broad range of disciplines is essential. Depending on circumstances and available resources, the multidisciplinary team should include the patient, diabetes specialist, primary care physician, nurse, dietitian, podiatrist and psychologist/psychiatrist, as well as family and friends. All members of the team should work together to ensure continuity of care. Communication and coordination within the team are also imperative to ensure that all members share and are working towards the same treatment targets and recommendations.

***Recommendation: Adopt a multidisciplinary team approach with shared goals and recommendations*.**

## Conclusion

While studies such as DCCT/EDIC have helped inform and improve diabetes management, gaps in care still remain, with glycaemic – as well as cardiovascular – targets still not being met by a considerable proportion of patients. We hope the recommendations presented here by the *Global Partnership for Effective Diabetes Management* provide guidance on where gaps remain and how to address them based on recent evidence. As mentioned above, the management of type 1 diabetes is complex for both patients and health professionals, and it is through the multidisciplinary team that these recommendations can be best implemented.
